# Introducing the sporobiota and sporobiome

**DOI:** 10.1186/s13099-017-0187-8

**Published:** 2017-06-30

**Authors:** George Tetz, Victor Tetz

**Affiliations:** Human Microbiology Institute, 423 West 127 Street, New York, NY 10027 USA

## Abstract

Unrelated spore-forming bacteria share unique characteristics stemming from the presence of highly resistant endospores, leading to similar challenges in health and disease. These characteristics are related to the presence of these highly transmissible spores, which are commonly spread within the environment and are implicated in host-to-host transmission. In humans, spore-forming bacteria contribute to a variety of pathological processes that share similar characteristics, including persistence, chronicity, relapses and the maintenance of the resistome. We first outline the necessity of characterizing the totality of the spore-forming bacteria as the sporobiota based on their unique common characteristics. We further propose that the collection of all genes of spore-forming bacteria be known as the sporobiome. Such differentiation is critical for exploring the cross-talk between the sporobiota and other members of the gut microbiota, and will allow for a better understanding of the implications of the sporobiota and sporobiome in a variety of pathologies and the spread of antibiotic resistance.

The unique characteristics of spore-forming bacteria are conditioned by the presence of endospores that are practically impermeable due to various traits, including the components of the core, cortex, coat and membranes [1].

The resistance of these spores results in their long-term survival under unfavorable conditions and various types of environmental exposure, such as high and low temperatures, UV, radiation, nutrient and water deprivation, antimicrobial agents, and the host immune system [2]. Such resistance results in the broad spreading of spore-formers in the outer environment, high transfer rates between various distinct ecological niches and frequent exchange between living organisms, including humans, in whom we suggest that spore-formers play a particular role in the microbiota [3, 4].

The effectiveness of microbiota research is influenced by the source from which it isolated (e.g. environmental microbiota, human microbiota, gut), as well as the components of the microbiota. Thus, to elucidate the bacterial components of microbiota, bacterial microbiomes are studied; viral or fungal components are investigated as part of virome or mycobiome research, while the determination of the collection of all antibiotic resistance genes requires the study of the resistome [5–8]. Such differentiation is important for exploring the interplay between these various components of the microbiota and to better understand the particularities of their individual impacts on the host.

We first outline the necessity of describing spore-forming bacteria as a separate entity within the global ecosystem. Then, we summarize the major unique characteristics of spore-formers, especially those related to human health—particularly to the gut—and discuss opportunities for future research.

## The sporobiota and the sporobiome

We identified traits that allow the identification of endospore-forming bacteria as an independent functional group within global microbiota (Table 1).

**Table 1:** Unique common characteristics of endospore-formers related to the presence of highly resistant spores

Specifically influenced by natural selection
Have an individual arrangement for fitness costs of antibiotic resistance
Resistant to various physico-chemical treatments, including antibiotics
Have strong binding properties
Highly transmissible
Implicated in the spread of antibiotic resistance
Spores trigger host immune responses with detrimental effects
Infections caused by sporeforming bacteria share similar characteristics: persistence, chronicity, relapses

**Fig. 1:**
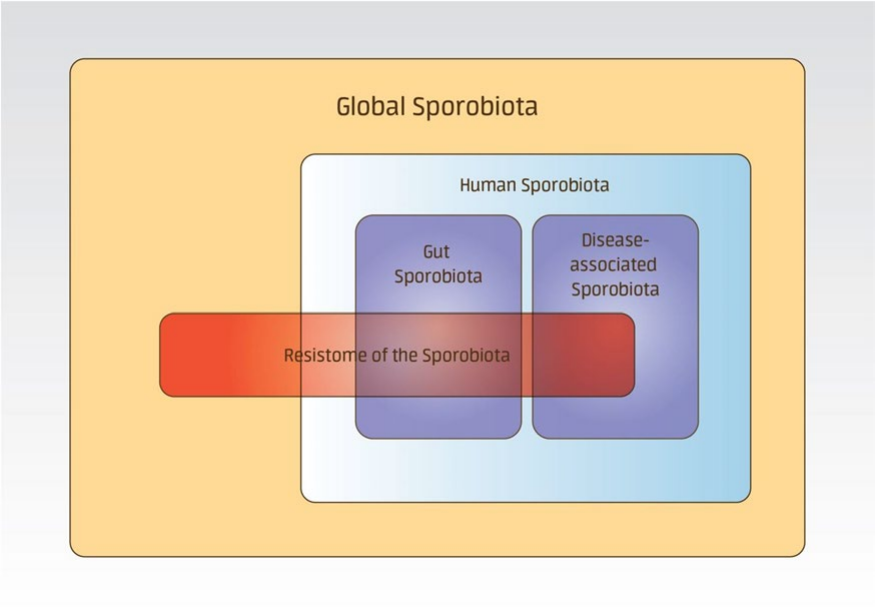
Conceptual presentation of the sporobiota. The *boxes* represent different levels of possible sporobiota representation. The global sporobiota comprises the totality of all endospore-forming bacteria. Both the human microbiota and the disease-associated sporobiota (clinical sporobiota) are subsets of the total global sporobiota. The global sporobiota, human sporobiota, disease-associated sporobiota, gut sporobiota and the resistome overlap, as spore-formers play a role in horizontal gene transfer

We suggest that the collection of all spore-forming bacteria be referred to as the sporobiota. To extend the functional analysis of the sporobiota, it can be can be classified based on different levels of representation, from the global sporobiota to the clinical (disease-associated) sporobiota of specific organisms or organ systems such as gut sporobiota (Fig. 1).

Similarly, we propose that the collective genomes of all genes of spore-forming bacteria related to a specific ecological niche be referred to as the sporobiome, which can be divided into different subgroups in accordance with the sporobiota’s identification.

Taking into consideration the unique characteristics of spore-forming bacteria analyzed in the present study, we suggest that the differentiation (identification) of spore-formers as sporobiota and the collection of all their genes into a sporobiome can shed new light on their particular interplay with other members of the microbiota.

Notably, the study of the sporobiota and the sporobiome as independent components of the microbiota and the microbiome will facilitate the identification of their individual impacts on human health including gut health. Furthermore, these studies may aid in the elucidation of the specific roles of the sporobiota and the sporobiome in horizontal gene transfer between ecological niches and their contribution to the spread of antibiotic resistance.

An emerging factor in characterizing the sporobiota and sporobiome independently is that due to advances in technology, we now understand that the true role of endospore-forming bacteria is poorly studied because the investigation of their biodiversity remains challenging [3, 9, 10].

The traditional approaches for determining the diversity of spore-forming bacteria in environments and hosts are challenging due to technical and biological difficulties. One of the challenges involved is that endospores are resilient to many traditional methods of DNA isolation and are therefore potentially undetectable [4]. Another challenge is the high similarity between 16S rRNA and housekeeping genes among unrelated spore-formers, which lead to underestimation in metagenomic analyses [11, 12]. In addition, spore-forming bacteria exhibit a larger average genome size than non-spore-formers, resulting in fewer reads per gene per taxon and leading to lower abundance estimates in metagenomic analyses [13–15].

**Fig. 2:**
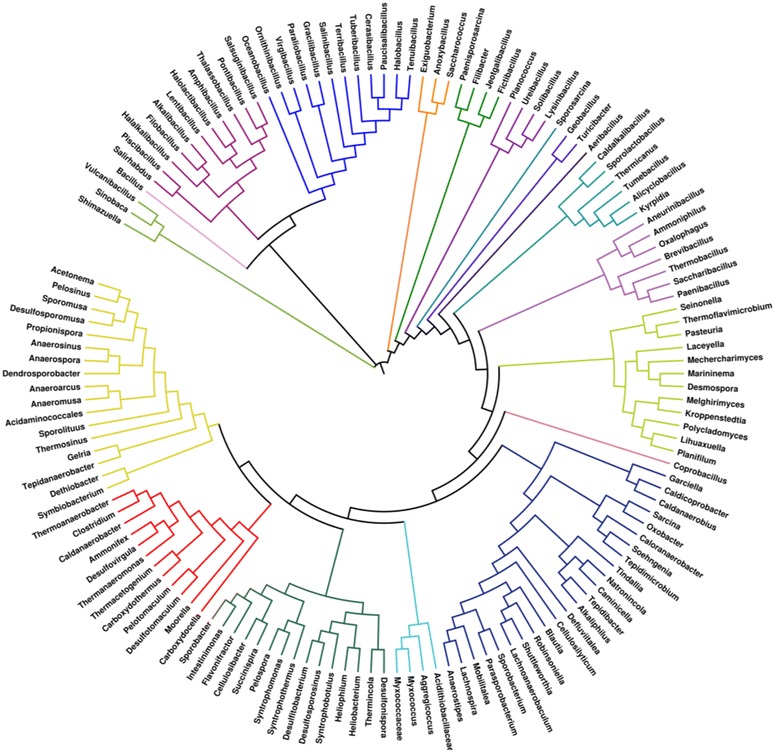
Phylogenetic representation of global sporobiome families based on 16S sequence similarity. Alignment was performed with CLUSTAL W. The visualization of phylogenetic trees and statistical analyses were performed using iToL [18]

Genomic-based workflows using combined genetic, culture and metagenomics methods are implemented for targeting endospore-forming bacteria and allow the identification of novel families, genera and species in the environment and human microbiome [4, 16, 17] (Fig. 2).

Some of these bacteria recently identified in the human microbiome have never previously been identified in humans or are representatives of previously unknown species, including species contributing to diseases [19]. In agreement with Celandroni et al., we identified a number of previously unknown spore-formers (including species isolated from the human gut) associated with human pathology as representatives of the disease-associated (clinical) sporobiota [9, 11].

We further summarize the major unique characteristics of spore-formers, especially those related to human health.

## Clinical challenges related to sporobiota: persistence and relapses

In addition to recently isolated spore-forming bacteria, there are several endospore-forming species associated with the human gut microbiome. The most prominent anaerobic bacterial spore-formers in the healthcare environment are members of the *Clostridiaceae* family and they are the causative agents of a variety of diseases, including tetanus, gas gangrene, and botulism [20]. *Clostridium difficile* causes a variety of intestinal diseases, from mild diarrhea to severe life-threatening inflammation of the colon [21]. Members of the *Lachnospiraceae* are abundant in the digestive tracts of mammals and have been linked to obesity, whereas colonization by members of the *Erysipelotrichaceae* is associated with gut disorders such as inflammatory bowel diseases and colorectal cancer [22, 23].

Aerobic endospore-forming bacteria are also implicated in human health. The members of the *Bacillaceae* family are well-known representatives of aerobic endospore-forming bacteria isolated from the human gut. While *Bacillus anthracis* was the first member of this group to be associated with human pathology, *Bacillus cereus* is currently recognized as being associated with human diseases such as endocarditis, diarrhea, and irritable bowel syndrome, in addition to being a pathogen associated with traumatic wounds and burns [24].

*Paenibacillus* spp. bacteria were not known to cause human disease until recent reports implicated *P. alvei*, *P. thiaminolyticus*, and *P. sputi*, *Paenibacillus* sp. VT-400 in bloodstream, respiratory and urinary tract infections [9, 25, 26].

Persistence within the host is an important characteristic of spore-formers and is a common characteristic of disease processes caused by these bacteria [27, 28].

A major challenge contributing to persistence is that spores are metabolically dormant and show reduced susceptibility to biocides, thus playing an important role in disease chronicity [29].

Notably, most infections are attributed to the biofilm mode of growth, which is associated with reduced susceptibility to antibiotics, and microbial communities formed by spore-forming bacteria are particularly resistant to antimicrobial therapy [30].

The same characteristics contribute to the high relapse rate of infections caused by spore-forming bacteria; after the death of vegetative cells, spores allow bacterial regrowth [31]. Thus, recurrent infection after the cessation of antibiotic therapy is a hallmark of spore-forming bacterial persistence [32, 33]. Numerous reports have shown that during the treatment of infections caused by *Clostridium* spp., a few days after antibiotic withdrawal, animals exhibited recurrent infection with the same isolate (i.e., relapse) [34]. In clinical practice, the recurrence of symptoms after initial improvement of infections caused by *Clostridium* spp. is known to be as high as 35% [27]. Based on the common evolutionary and phenotypic characteristics of sporulation and germination, other spore-formers rely on the same interplay with the host as *Clostridiales* species [31]. Similarly, infections caused by members of the order *Bacillales* are characterized by relapses, with restoration of bacterial growth after successful antibiotic treatment followed by the clinical improvement [35].

Another challenge associated with members of the sporobiota is that spore-associated elements, such as nucleic acids of the core or core proteins, trigger host immune responses with detrimental effects [36]. Taken together, these data suggest that unrelated spore-forming bacteria exert certain effects on the host that are nearly identical.

## Transmission and spreading of the sporobiota and sporobiome

The robustness of spores is attributed to their resistance to various physio-chemical treatments, including most disinfectants, in addition to their strong binding properties. As such, spores are the primary agents of the rapid dispersal-conditioned global spreading of endospore-formers [1, 37].

Spore-forming bacteria exhibit unique survival strategies that facilitate their dispersal to and subsequent colonization of new locations; thus, they play a unique role in gene flow and horizontal gene transfer [38–40].

Spore-forming bacteria are globally distributed, including in extreme environments. Existing metagenomics methods indicate that in the outer environment, endospore-formers are relatively rare, with an abundance of more than 1% being observed only for *Bacilli* and *Clostridia* [38]. However, recent studies have indicated that the numbers of spore-formers in the environment and in hosts are underestimated due to the technical challenges of DNA isolation from endospores [3, 4].

The transmission potential of members of the sporobiota and sporobiome plays an important role in human health and diseases. First, spores are specialized for host-to-host transmission. This property has important implications for the inheritance of the microbiota and its colonization of the infant gastrointestinal tract [3, 41]. Spore-formers disseminate through patient-to-patient contact, playing a role in host-to-host transmission, which is expected to facilitate nosocomial infections [31, 42]. Consequently, health care workers and patients, even as asymptomatic carriers, are important vectors for the nosocomial transmission of spore-forming bacteria.

Environmental spore-forming bacteria that produce spores (including those found in soil) are constantly introduced into the human microbiota, as they more aero-tolerant than non-spore-formers [3]. Such bacteria represent a risk to immunocompromised hosts, in whom the environmental sporobiota constitutes an important source of potential infection [43]. Spore-forming bacteria survive for long periods on medical surfaces and medical surroundings, making them an important source of patient contamination [44]. The same process allows for a vice versa transfer of spores from human to the outer environment leading to the accumulation of resistant spores that harbor multiple antibiotic-resistant genes in the human surroundings.

## The sporobiome and resistome

In the aggregate, the transmission of spore-forming bacteria has implications for the spread of antimicrobial resistance and the transfer of resistance genes from the environmental resistome into human commensals and pathogens. Bacterial spores contain a complete copy of the genome and represent a pool of virulence and antibiotic resistance genes that may be implicated in the horizontal transfer of resistance genes to other bacterial species [45, 46].

We have previously identified many antibiotic resistance genes in a number of novel species of spore-forming bacteria isolated from the human gut [9, 11, 16]. We found that some of these resistance genes are not expressed; however, unexpressed genes remain a component of the resistome, from which pathogenic bacteria can acquire resistance via horizontal gene transfer [47].

Recent studies have also shown that environmental spore-forming bacteria exhibit an extremely high diversity of antibiotic resistance genes.

Taking into consideration high transfer rates and apparent overlap between different ecological niches of the members of sporobiota including overlap between environmental and clinical species, this represents a particular challenge for the spread of antibiotic resistance [5, 48, 49]. The sporobiome serves as a unique reservoir for the dissemination of resistance that has already emerged or has the potential to emerge in clinically important bacteria [50]. It is therefore vital that the resistance properties of spores and their high transmission potential are employed to delineate the unique role of the sporobiome in the resistome.

## Conclusions and perspectives

In this article, we propose the new terms sporobiota and sporobiome, highlighting that this distinction is not solely an academic issue. Based on specific, common characteristics of endospore-formers compared with other representatives of the microbiota, such a differentiation has important implications, predominantly for human health and diseases. These characteristics are related to the presence of highly transmissible spores, which are commonly spread within the environment and are implicated in host-to-host transmission. Such similar characteristics of the pathological process include a chronic and recurrent course, the specific immune response to the spores, and the spreading and maintenance of the resistome.

We first outlined how such a distinction will reveal the particularities of the interaction of the sporobiota with other ecosystem components of the environmental and human microbiota, which will facilitate the identification of new disease treatments and ways to overcome the spreading of antimicrobial resistance.

The high transmission dynamics and geographic distribution of spore-formers are well documented. However, the study of sporobiota biodiversity will require improvements in molecular tools. Existing genetic methods, such as high-throughput sequencing, have insufficient analytical accuracy for the elucidation of biodiversity and do not allow the evaluation of the true abundance of spore-formers in the environment or hosts. Further research is likely to be concentrated on the improvement of computing power, the development of specific isolation workflows combining traditional culture approaches with genetic strategies and the creation of specific databases. Using developed workflows, we have only just begun to understand the high level of diversity that has been revealed and to just scratch the surface of the sporobiota and sporobiome. Much more work remains.
